# Surface pH changes suggest a role for H^+^/OH^−^ channels in salinity response of *Chara australis*

**DOI:** 10.1007/s00709-017-1191-z

**Published:** 2017-12-15

**Authors:** Marketa Absolonova, Mary J. Beilby, Aniela Sommer, Marion C. Hoepflinger, Ilse Foissner

**Affiliations:** 1 Department of Cell Biology and Physiology/Plant Physiology, University of Salzburg, Salzburg, Austria; 2 School of Physics, The University of NSW, Sydney, NSW 2052 Australia

**Keywords:** Salinity stress, Plant H^+^/OH^−^ channels, Fluorescent pH measurement, *Chara*, Internodal cell, pH banding

## Abstract

To understand salt stress, the full impact of salinity on plant cell physiology has to be resolved. Electrical measurements suggest that salinity inhibits the proton pump and opens putative H^+^/OH^−^ channels all over the cell surface of salt sensitive *Chara australis* (Beilby and Al Khazaaly 2009; Al Khazaaly and Beilby 2012). The channels open transiently at first, causing a characteristic noise in membrane potential difference (PD), and after longer exposure remain open with a typical current-voltage (I/V) profile, both abolished by the addition of 1 mM ZnCl_2_, the main known blocker of animal H^+^ channels. The cells were imaged with confocal microscopy, using fluorescein isothiocyanate (FITC) coupled to dextran 70 to illuminate the pH changes outside the cell wall in artificial fresh water (AFW) and in saline medium. In the early saline exposure, we observed alkaline patches (bright fluorescent spots) appearing transiently in random spatial distribution. After longer exposure, some of the spots became fixed in space. Saline also abolished or diminished the pH banding pattern observed in the untreated control cells. ZnCl_2_ suppressed the alkaline spot formation in saline and the pH banding pattern in AFW. The osmotic component of the saline stress did not produce transient bright spots or affect banding. The displacement of H^+^ from the cell wall charges, the H^+^/OH^−^ channel conductance/density, and self-organization are discussed. No homologies to animal H^+^ channels were found. Salinity activation of the H^+^/OH^−^ channels might contribute to saline response in roots of land plants and leaves of aquatic angiosperms.

**Electronic supplementary material:**

The online version of this article (10.1007/s00709-017-1191-z) contains supplementary material, which is available to authorized users.

## Introduction

The motif of spatially separated proton pumps and H^+^/OH^−^ channels generating acid and alkaline zones can be found in many branches of plant kingdom. The roots of land plants display subapical acidification and apical alkalinization with H^+^ or OH^−^ circulating currents to improve molybdenum, phosphorus, and iron acquisition and reduce aluminum toxicity (Raven 2000, 1991). The H^+^/OH^−^ channel current-voltage (I/V) characteristics have been modeled in wheat root protoplasts (Tyerman et al. 2001). The mechano-sensing in roots also seems to involve rapid pH changes (Monshausen et al. 2009). The apical acid zone oscillates in pollen tubes and together with Ca^2+^ fluxes aids growth (Feijo et al. 1999; Michard et al. 2008). The aquatic angiosperms (Prins et al. 1980; Lara et al. 2002) and Characeae (Spear et al. 1969; Lucas and Smith 1973; Walker and Smith 1977; Ray et al. 2003) employ acid and alkaline zones (pH banding) as a biophysical carbon concentrating mechanism to increase carbon assimilation and photosynthetic efficiency (Raven and Beardall 2016; Raven and Hurd 2012). The exact nature of the carbon import into characean cells is yet to be worked out: CO_2_ and H_2_CO_3_ diffusion, HCO_3_^−^ transport, or both are possible in the acid bands (Walker and Smith 1977; Lucas 1982). Upon sufficient illumination, the banding pattern forms within minutes (see Beilby and Bisson 2012 for review) with the pH increase in the alkaline bands by several pH units (Bulychev et al. 2001). The banding cells set up a pattern of circulating currents, with positive charges leaving the cell in the acid regions and entering in the alkaline regions, supporting the H^+^ pump and H^+^/OH^−^ channel combination (Walker and Smith 1977, Lucas and Nuccitelli 1980, Dorn and Weisenseel 1984).

The hypothesis that H^+^/OH^−^ channels exist in Characeae plasma membrane is also supported by the “high pH state” (Bisson and Walker 1980). When the pH of the outside medium, pH_o_, is buffered to values above 9, the whole *Chara* cell becomes an alkaline band and the membrane PD follows the equilibrium (Nernst) PD for H^+^ or OH^−^, E_H_/E_OH_, up to pH_o_ 11.5 (Bisson and Walker 1980, 1981). Consequently, there is no electrochemical gradient to power any symporters or antiporters with H^+^. The ambiguous name of the H^+^/OH^−^ channels arises from the difficulty of distinguishing between H^+^ and OH^−^ conduction (DeCoursey and Hosler 2014). Beilby and Bisson (1992) measured current voltage (I/V) characteristics of high pH state and found conductances of up to 10 S m^−2^, impossible to achieve with few H^+^ in the medium. Lucas (1979) also concluded that OH^−^ is probably the transported ion. We continue to refer to the characean channels as “H^+^/OH^−^,” as the defining patch clamp measurements have yet to be performed. For comprehensive reviews of the high pH state and pH banding in Characeae see Chap. 2 of Beilby and Casanova (2014) and Beilby and Bisson (2012).

The H^+^/OH^−^ channels are well researched and genetically characterized in animal systems. The transport occurs by defect propagation along water molecules arranged in a single file (water wire) in the channel core: Grotthuss mechanism (H^+^) or a proton “hole” migration (OH^−^) (see Fig. 5 in DeCoursey and Hosler 2014). For instance, the human proton channel H^+^ conductivity is determined by a specific location of negatively charged aspartic acid residue (Asp^112^). The replacement of Asp^112^ by a neutral amino acid such as Ser, Ala, or Asn results in anion selectivity of the channel (Musset et al. 2011). The animal H^+^ channels are voltage-gated and their opening is favored by depolarization of membrane potential difference (PD). The gating is also dependent on ΔpH (pH_o_–pH_i_) (DeCoursey 2013). The characean H^+^/OH^−^ channels in the high pH state are activated by an increase in pH_i_ or by pH_o_ above 9 (Bisson and Walker 1980, 1981) and by negative PDs (Beilby and Bisson 1992).

**Fig. 1:**
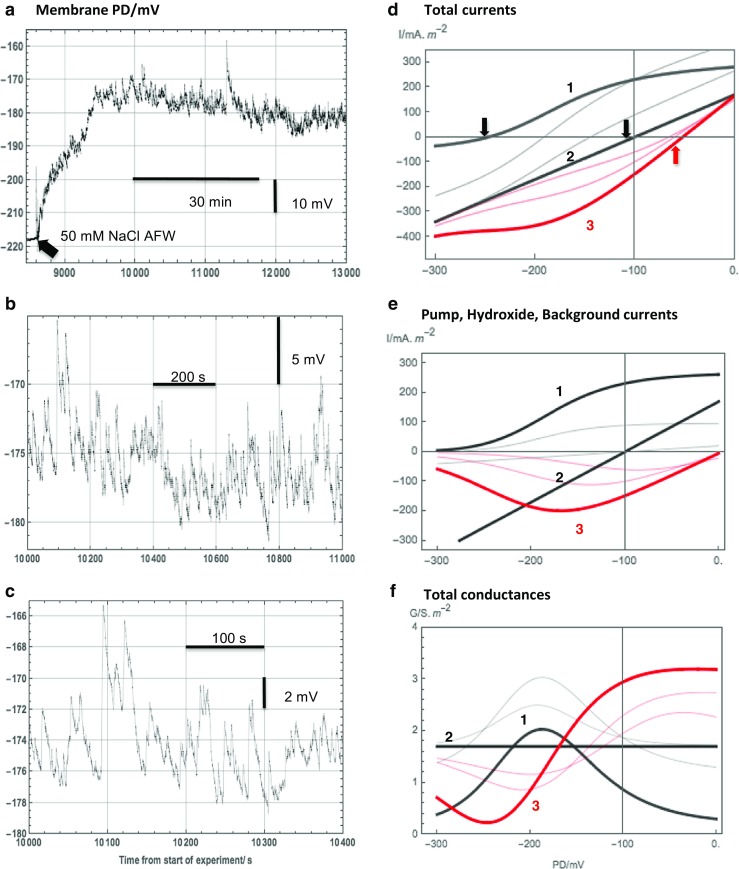
**a**–**c** The salinity-induced noise in membrane potential difference (PD). **a** The membrane PD promptly depolarized and became noisy upon exposure to saline AFW. **b** and **c** The noisy PD on different time scales. The readers are reminded that the electrical measurement integrates the noise all over the cell surface. **d**–**f** The evolution of current-voltage (I/V) and conductance-voltage (G/V) characteristics before and after exposure to saline AFW. This simulation is based on data from many experiments with the inward rectifier current omitted for clarity (see Beilby and Al Khazaaly 2009 for typical model parameters). The timing of the different stages, numbered 1–3, varies from cell to cell. **d** Total currents: 1 the pump current and background current in sorbitol AFW, 2 the background current with greater conductance enhanced by the exposure to saline AFW (Shepherd et al. 2008), 3 the OH^−^ current and the background current after longer exposure to saline AFW (red trace). The I/V curves shown with thinner lines represent the transition from the pump-dominated state to the background state (black) and to the OH^−^ channel dominated state (red). The arrows designate the resting PDs for each main state: pump state, − 247 mV, background state, − 100 mV, OH^−^ state, − 52 mV. **e** The component currents: 1 the pump current in sorbitol AFW, 2 the more conductive background current enhanced by exposure to saline AFW, 3 the OH^−^ current after longer exposure to saline AFW (red trace). The I/V curves drawn with thinner lines show the background current in the sorbitol AFW, the decline of the pump current in the saline AFW (black), and the increased participation of the OH^−^ channels (red). **f** The conductances calculated from the I/V characteristics of **d**. The readers are reminded that the conductance is the slope of the I/V curve and that the parallel conductances are additive. Note that the maximum OH^−^ conductance is initially near − 100 mV and extends to more negative PDs with longer exposure to saline AFW. In the electrophysiological experiments, the *Chara* cells were allowed to recover after electrode impalement in AFW for about an hour, then the sorbitol was added to AFW (sorbitol AFW) to condition the cell to saline shock. After another hour, cells were exposed to saline AFW. The measurements in this figure are single examples from previously published large data sets (Al Khazaaly et al. 2009; Beilby and Al Khazaaly 2009; Beilby et al. 2014). For detailed Fourier analysis of the noise spectra see Al Khazaaly et al. (2009)

In salt sensitive Characeae *Chara australis*, the transporters participating in the cell pH banding pattern are strongly affected by salinity stress. Comparatively mild salinity of 50–100mM NaCl increases the background conductance and inhibits the proton pump within hours of exposure, if the Ca^2+^ concentration is low at 0.1 mM (Shepherd et al. 2008). Within seconds of exposure to saline media, the membrane PD exhibits a typical noise (Al Khazaaly et al. 2009 and Fig. 1a–c). The authors hypothesized that groups of H^+^/OH^−^ channels open spontaneously and transiently all over the cell surface (Al Khazaaly et al. 2009; Beilby et al. 2014). With time in saline medium, the cell PD becomes strongly depolarized to levels above − 100 mV, the PD noise diminishes, and the I/V characteristics can be modeled with the H^+^/OH^−^ channels (Beilby and Al Khazaaly 2009 and Fig. 1d–f). With these channels as the dominant transporter, the membrane PD approaches 0 and the cytoplasm grows more acid (Katsuhara et al. 1989). The Na^+^/H^+^ antiporter, vital to keep the cytoplasmic Na^+^ low, is no longer powered by the proton gradient. This hypothesis of cell sabotage by the global opening of H^+^/OH^−^ channels was further supported by application of ZnCl_2_, the main known blocker of animal H^+^ channels (DeCoursey 2003; Musset et al. 2010). The high pH state, the pH banding, the PD noise, and the latter depolarization and H^+^/OH^−^ I/V characteristics in salt stressed cells were all abolished by 1 mM ZnCl_2_ (Al Khazaaly and Beilby 2012). However, in plants, Zn^2+^ also blocks aquaporins (Rygol et al. 1992; Tazawa et al. 1996; Przedpelska-Wasowicz and Wierzbicka 2011) and SV channels (Pottosin and Schonknecht 2007; Hedrich and Kurkdjian 1988). Thus, the effects of ZnCl_2_ on the H^+^/OH^−^ channels were assessed with their multiple actions in mind (Al Khazaaly and Beilby 2012).

How does salinity stress activate the H^+^/OH^−^ channels? The saline media are several pH units below the pH activation threshold (Bisson and Walker 1981). Eremin et al. (2013) used fluorescent probe dihydrodichlorofluorescein (DCHF) to map out the reactive oxygen species (ROS) formation under strong spot illumination of *Chara* surface: ROS from the chloroplasts moved with the cytoplasm, possibly oxidizing sulfhydryl (SH) groups on the H^+^/OH^−^ channel proteins and leading to their opening. The regions of the cell with most intense fluorescence exhibited greatest pH increase on the outside. On the other hand, exposure of *Chara* cell to DTT (dithiothreitol) diminished the alkaline regions. A closure of H^+^/OH^−^ channels by the reduction of disulfide bonds was assumed (Dodonova et al. 2010). The pretreatment of *Chara* cells in potent antioxidant melatonin delayed the appearance of the saline noise upon exposure to saline stress (Beilby et al. 2014). The authors did measure ROS concentrations, but the temporal and spatial resolution of the measurements was not sufficient to correlate it with the noise events. Li et al. (2007) measured a rapid oxidative burst upon exposing rice roots to saline media. It is possible that in giant Characeae cells, oxidative events occur in different parts of the cell, activating the channels. ROS signaling and pH changes feature in mechano-sensing in *Arabidopsis* roots, but their connection is also unclear (Monshausen et al. 2009). In the animal kingdom, the H^+^ channels in the brain microglia are activated by H_2_O_2_ (Wu 2014). So, while ROS signaling is implicated in activation of H^+^/OH^−^ channels in salt stressed cells, more research is necessary.

The electrophysiology, modeling, and Zn^2+^ block are not sufficient to prove the identity of the channel entity involved in *Chara* saline stress. We hypothesized that the transient (and later more persistent) opening of the H^+^/OH^−^ channels should result in changes of pH in the medium around the cell undergoing saline stress. In the present study, we employ fluorescein isothiocyanate (FITC) conjugated with 70 kDa dextran to illuminate the pH changes outside the cell wall in artificial fresh water (AFW), sorbitol AFW (which simulates the osmotic component of the saline), and in 50 mM Na^+^/0.1 mM Ca^2+^ saline AFW.

## Materials and methods

### Algal material and culture conditions

The cultures of *C. australis* were grown at about 20 °C in 10 L tanks in a substrate of soil, peat, and sand covered by distilled water. The tanks were illuminated by fluorescent lamps providing light intensity of 5 μ Einstein m^−2^ s^−1^ at the water surface and operating on a 14/10 day/night cycle. The internodal cells of branchlets were cut and stored in AFW (1 mM NaCl, 0.1 mM KCl, 0.1 mM CaCl_2_, unbuffered pH 5.6) several hours prior to the experiment.

### Confocal laser scanning microscopy (CLSM) and image processing

The internodal cells of *Chara* branchlets were placed into AFW, supplemented with 10 μM FITC-dextran 70 (fluorescein isothiocyanate-dextran 70 kDa; Sigma-Aldrich, St. Louis, MO, USA). The cell wall was not accessible to FITC because of the attached 70 kDa dextran (Berestovsky et al. 2001; Proseus and Boyer 2005); so, all the pH changes were observed in the external medium. A control scan was made for each cell. The cytoplasmic streaming was monitored throughout the experiments. The bath solution was then exchanged for AFW supplemented with 10 μM FITC-dextran 70 and with 50 mM NaCl (saline AFW), in order to visualize the pH changes evoked by the saline exposure. In some experiments, the cells were exposed to AFW with 90 mM sorbitol (sorbitol AFW) to distinguish the osmotic and saline stress components. The sorbitol was used, as it was previously employed in the electrophysiological experiments, where it produced no changes in the I/V characteristics (Shepherd et al. 2008; Beilby and Al Khazaaly 2009). In final experiments, 1 mM ZnCl_2_, a potent blocker of animal H^+^ channels, was added to AFW and saline AFW. The Zn^2+^ concentration of 1 mM is high compared to many animal system experiments, but this concentration is occasionally used (Cherny and DeCoursey 1999; Asuaje et al. 2017). Up to 5 mM Zn^2+^ was used in *Chara* aquaporin blockage experiments (Rygol et al. 1992).

The confocal laser scanning microscope used in this study was a Leica (Mannheim, Germany) TCS SP5 coupled to a DMI 6000B inverted microscope. Images were taken with a ×10 objective (numerical aperture 0.4).

FITC can be used as a ratiometric dye in order to correct the signal for unequal dye loading and bleaching or as a non-ratiometric dye. We chose the latter method because of the following reasons. Firstly, we measured the extracellular pH, where the dye is not compartmentalized; secondly, the bleaching of FITC was negligible under the conditions applied in this study; thirdly, the calibration curve of FITC is linear between pH 5 and 7.5 and then becomes comparatively flat (Lanz et al. 1997). Therefore, it is not possible to determine the exact value of pH in the alkaline bands and spots of *Chara*, which have a pH of 7.5 and higher. The dye can be used, however, to show pH increases and decreases relatively to the background in the medium along the internodal cell, provided that they are within the detection range. For this purpose, the fluorescence of FITC-dextran 70 was excited with the 488 nm line of the argon laser and detected in the range 500–575 nm. Time series (1.2 second interval between frames) were taken with durations of up to 45 min, and Z-stacks with a step size of 1.3 μm were recorded to map the fluorescent spots over about 18% of the cell surface in the scanned area (compare Fig. 4). All the images were processed using Leica software and ImageJ (http://imagej.nih.gov/ij). For details see figure legends. The kymograph in Fig. 4c was obtained as follows: the time series was converted into a series of 32 bit gray scale images and was subsequently resliced, thus creating a Z-stack with the time being encoded along the z-axis. The kymograph was generated as the maximum intensity projection of this stack.

### Sequence analyses

Total RNA was extracted from *C. australis* thalli and transcriptomic data were obtained on the one hand from a normalized random primed cDNA library followed by 454 sequencing (Roche GS FLX system; Eurofins MWG) and on the other hand from a normalized cDNA library sequenced by Illumina Hiseq 2000 Technology (BGI Genomics, Hong Kong, China). Different annotated plant voltage-gated hydrogen channel (VGHC) sequences, voltage sensing domains, and the human VGHC (HVCN1, accession number Q96D96) were used for BLAST analyses (Altschul et al. 1990) in order to reveal homologous proteins in our *C. australis* databases. The conserved domains were detected using InterPro (Mitchell et al. 2015).

Furthermore, the different VGHCs of plants (*Nicotiana sylvestris* XP_009803685, *N. tabaccum* XP_00961780, *Fragaria vesca* XP_004298021, *Glycine soja* KHN29519, *Medicago truncatula* XP_003621655, *Klebsormidium flaccidum* GAQ80331, *Selaginella moellendorfii* XP_00298883, *Marchantia polymorpha* OAE32766, *Physcomitrella patens* XP_001767834) were aligned using ClustalOmega (EMBL-EBI) to define overlapping, homologous regions. These areas were screened for sequence parts suitable for the design of degenerated primers. The binding sites of these primers are indicated by blue bars in Suppl. Fig. 4b. Primer sequences are listed in Suppl. Table 1. The template cDNAs for regular and nested PCRs were prepared from thalli of *C. australis* under the control conditions as well as in response to NaCl stress (50-mM NaCl in AFW for 2.5 h). The total RNA was extracted using TRI-Reagent according to the manufacturer’s instructions (Sigma-Aldrich). The residual genomic DNA was digested using RNase-free DNase (EN0521, Thermo Fischer Scientific, Waltham, MA, USA) and first-strand cDNA was synthesized by M-MuLV Reverse Transcriptase (RevertAid; EP0441, Thermo Fischer Scientific) combined with an anchored oligo(d)T primer-mix according to the supplier’s protocol. The obtained cDNAs were used as templates for PCRs with either homemade Taq polymerase or KAPA3G Plant PCR kit (KAPA Biosystems, Sigma-Aldrich) according to the manufacturer’s instructions. All the primer combinations as well as the different PCR conditions were systematically tested using control as well as salt stress cDNAs as templates, annealing temperatures in the range of 40–60 °C, and extension times suitable for 400–1200 bp.

## Results

**Fig. 2:**
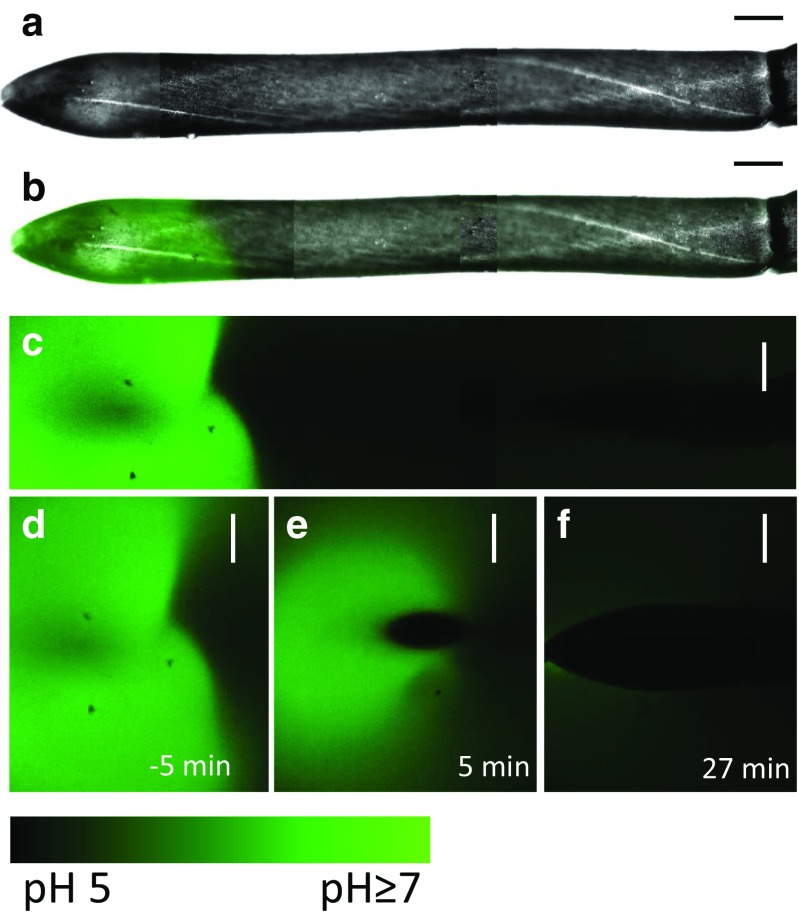
The inhibition of pH banding in saline-stressed *Chara* cell. **a**–**c** The alkaline band at the tip of a branchlet internodal cell was visualized with 10 μM FITC-dextran (**a** bright field image, **b** bright field image merged with fluorescence image, **c** fluorescence image). The dark region reflects an acidic pH. **d**–**f** Detail of the tip before **d** and after addition of saline AFW with 50 mM NaCl **(e**–**f)**. The images **a** and **b** are assembled from five overlapping optical sections along the cell. The white stripes are the chloroplast-free neutral lines, which separate opposing cytoplasmic streams. Bars are 250 μm

We first investigated the fluorescence of FITC-dextran in the extracellular medium surrounding the untreated internodal cells. In AFW the pH banding pattern was clearly visible upon addition of FITC-dextran 70 and intensified with time (a single band in the apical area of a leaf cell is shown in Fig. 2b and c). In some cells, the alkaline band could be observed forming from smaller bright spots coalescing (see the sequence in Suppl. Fig. 1a), possibly due to manipulation-induced changes in the channel conductivity. Some cells exhibited stationary (permanent) high pH spots during the recording period and these were associated with wound walls. The wound walls were not produced during the course of present study but were deposited already during the cultivation of plants either after local cell damage or as a response to epiphytic organisms (Suppl. Fig. 2; Foissner and Wasteneys 2012). The wound wall-associated alkalinization of the external medium has also been described using phenol red as pH indicator (Shimmen and Yamamoto 2002; Foissner et al. 2015). The addition of 1 mM ZnCl_2_ to AFW abolished banding (Suppl. Fig. 3) and the wound wall-associated alkalinization.

After the cells were exposed to saline AFW supplemented with FITC-dextran, the alkaline bands weakened/disappeared with different time courses for different cells (Fig. 2e and f, Suppl. Fig. 1b). This behavior was typical for most of the cells investigated. Out of 15 cells exposed to saline AFW, 13 cells lost their pH band(s) within minutes, whereas only in two cells the bands were still visible, but considerably reduced in size and intensity. Ninety percent of the FITC-imaged cells (*n* = 20) still showed cytoplasmic streaming after 48 h in saline AFW.

**Fig. 3:**
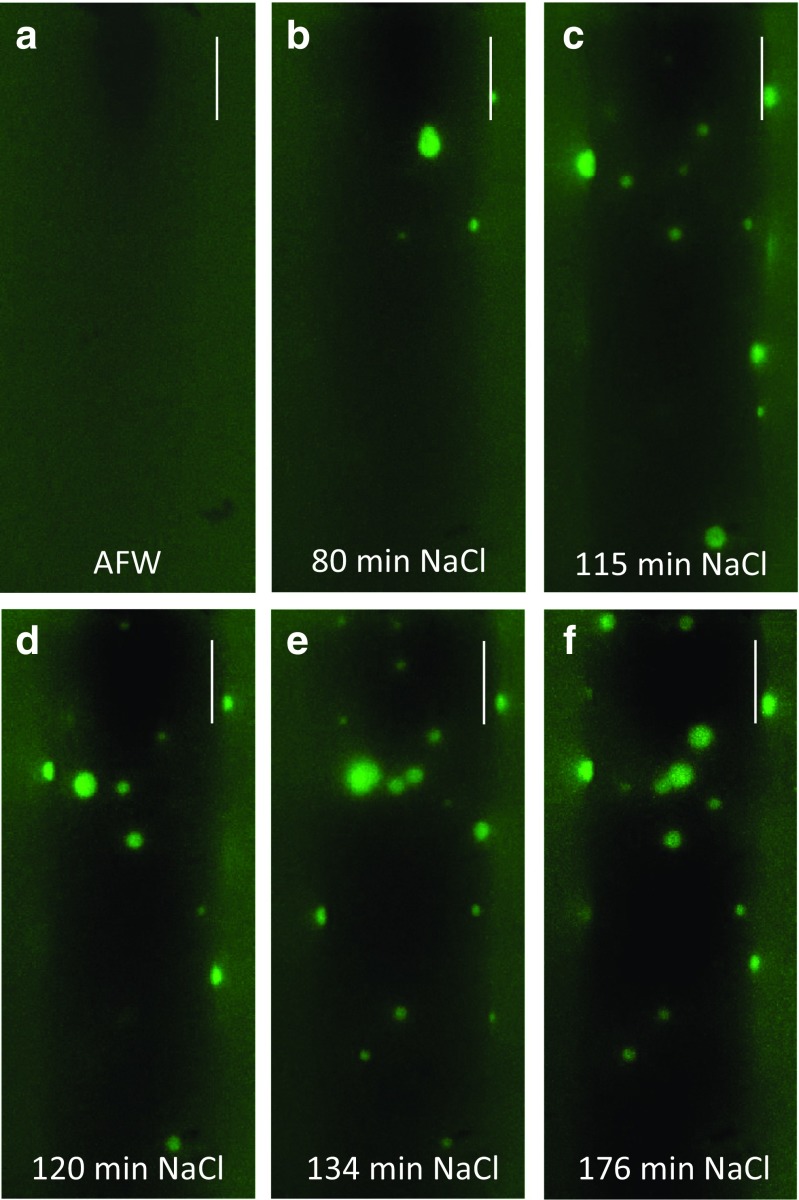
The alkaline spots appear at the cell wall surface of a salt-treated *Chara* cell. The pH was visualized with 10 μM FITC-dextran dissolved in AFW (control) or in 50 mM NaCl. The images are projections of Z-stacks taken before (**a**) and after (**b**–**f**) the introduction of NaCl. Each projection comprises 86–147 images, which corresponds to 18% of the cell surface. Bars are 200 μm

**Fig. 4:**
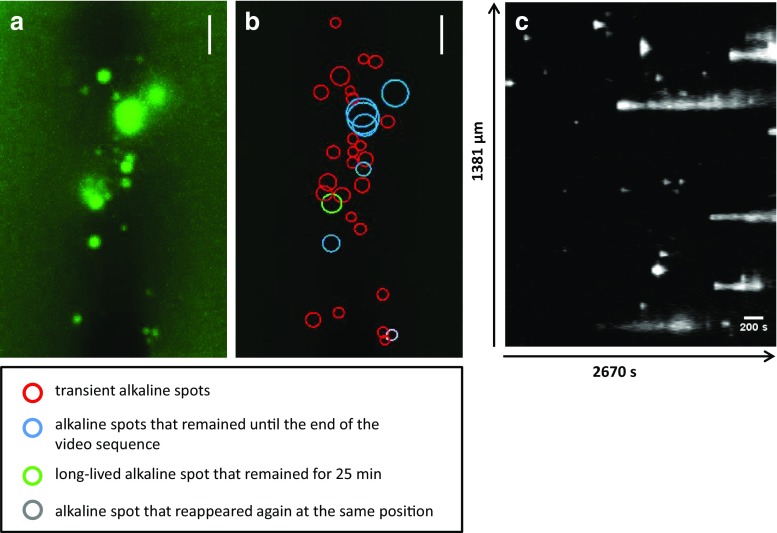
The dynamics of alkaline spots visualized by FITC-dextran. **a** Maximal projection of 2095 images of a time series taken 77–121 min after the introduction of saline AFW. **b** Lifetimes of alkaline spots are designated by different colors. **c** Kymograph of the same time series illustrating the life times of alkaline spots. Note that some of the transient spots appear suddenly, then decline gradually, whereas others display slow rising and decaying kinetics. Bars in **a** and **b** are 100 μm

Within 10–18 min (*n* = 11 cells) after salt addition, spots of bright fluorescence, indicating a localized increase in pH, started to appear randomly distributed over the imaged part of the cell (Fig. 3b–f; Suppl. Videos 1a and b). The kinetics of the growth and decline of these alkaline patches were complex (Fig. 4); some of them appeared and grew to maximum size within 25 seconds, then vanished gradually within 130 seconds, whereas others displayed slower rising (50 seconds) and decaying (190 seconds) times. After longer exposure to saline AFW, some of the spots persisted longer (Fig. 4b and c). When the saline AFW was replaced by AFW for 30 min, the high pH spots were no longer detectable and cells recovered the ability to band (see below) within further 30 min (ten cells investigated).

**Fig. 5:**
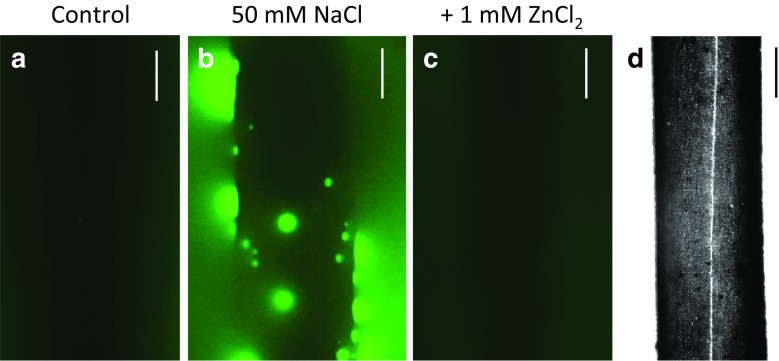
The maximal projections of Z-stacks (**a** 87, **b** 244, and **c** 132 images) from a region of a cell incubated in FITC-dextran, which was dissolved in AFW (**a**), then in saline AFW (**b**), and later in saline AFW + 1mM ZnCl_2_ (**c**); **d** is the corresponding bright field image. The white stripe in **d** is the chloroplast-free neutral line which separates up- and down-streaming endoplasm. Bars are 200 μm

Upon addition of 1 mM ZnCl_2_ to saline AFW, the number of spots greatly decreased and eventually disappeared within a few minutes (*n* = five cells) (Fig. 5).

In order to show that the effects described above were not due to a decrease in turgor, but to salinity stress, the pH around cells suspended in 90 mM sorbitol added to AFW (sorbitol AFW) was also monitored (*n* = 14). After 1 h in sorbitol AFW, the banding ability of the cells was not affected and dynamic, small spots were not formed.

In the sequence analysis, the different VGHC sequences, as well as their voltage sensing domains, were used as templates to search for homologous proteins in our transcriptomic databases of *C. australis* without success. In order to detect any VGHC sequences that might not be expressed in our databases, we compared different plant VGHCs to define an overlapping, homologous region for the design of degenerated primers (Suppl. Fig. 4). We systematically tested all the primer combinations (Suppl. Table 1), the different PCR conditions, and the different template cDNAs without useful outcome.

During our database screens, a member of the cation/H^+^ exchanger superfamily in *C. australis* was detected, which shares 40% amino acid identities with *A. thaliana* CHX17 (At4g23700) (Suppl. Fig. 5). Furthermore, we were able to find several partial protein sequences that showed 30–40% amino acid identity to various voltage-gated K^+^ channels of *A. thaliana*.

## Discussion

The initial reason for the present experiments was a confirmation of the hypothesis based on the electrophysiological data: upon exposure to saline AFW the H^+^/OH^−^ channels open transiently, causing typical membrane PD noise (Fig. 1a–c; Al Khazaaly et al. 2009; Beilby et al. 2014). After longer saline exposure, greater numbers of H^+^/OH^−^ channels remain open, becoming important in the I/V characteristics (Fig. 1d–f, Beilby and Al Khazaaly 2009). The experiments not only revealed pH changes around the salt-stressed cell but also highlighted the strengths of approaching a question with different techniques. Monitoring the pH changes in the vicinity of the cell provides information on permeability of the cell wall space, on dynamics of neutralization of the fixed charges on the wall, and also on the self-organization aspects of the H^+^/OH^−^ channels and the alkaline band formation and collapse.

In the electrophysiological experiments, the membrane PD noise started in a few seconds upon exposure to saline AFW (see Fig. 1a–c). However, the dynamic high pH spots (Fig. 3) appeared after ~ 15 min. The hydroxide ions, released by the transient opening of groups of H^+^/OH^−^ channels, are filtered through the cell wall, which is not accessible to FITC because of attached 70 kDa dextran (Berestovsky et al. 2001; Proseus and Boyer 2005). Let us assume that the passage of protons and hydroxide ions across the cell wall can be considered as Fickian diffusion in the bulk water, since the *Chara* cell wall pores have a diameter of ~ 4–5 nm (Proseus and Boyer 2005; Boyer 2009), much greater than the hydration radius of OH^−^ (~ 0.1 nm, Markus 2012). We can calculate the time *t* for the OH^−^ to cross the wall as 5 mseconds, with *t* ~ *d*^2^/2*D*, the wall thickness *d* = 6.5 μm (Kiyosawa and Adachi 1990) and the diffusion coefficient ${D}_{{\mathrm{OH}}^{-}}$ = 4.56 ± 1.29 *10^−5^ cm^2^ s^−1^ (Lee and Rasaiah 2011). Even increasing *d* by a factor of 10 due to the complex path through the tortuous wall pore geometry and decreasing *D* by a factor of 10 (an approximation made for small mobile molecules in the cell wall, Kramer et al. 2007), the diffusion time would be of the order of seconds, rather than the observed delay of ~ 15 min.

**Fig. 6:**
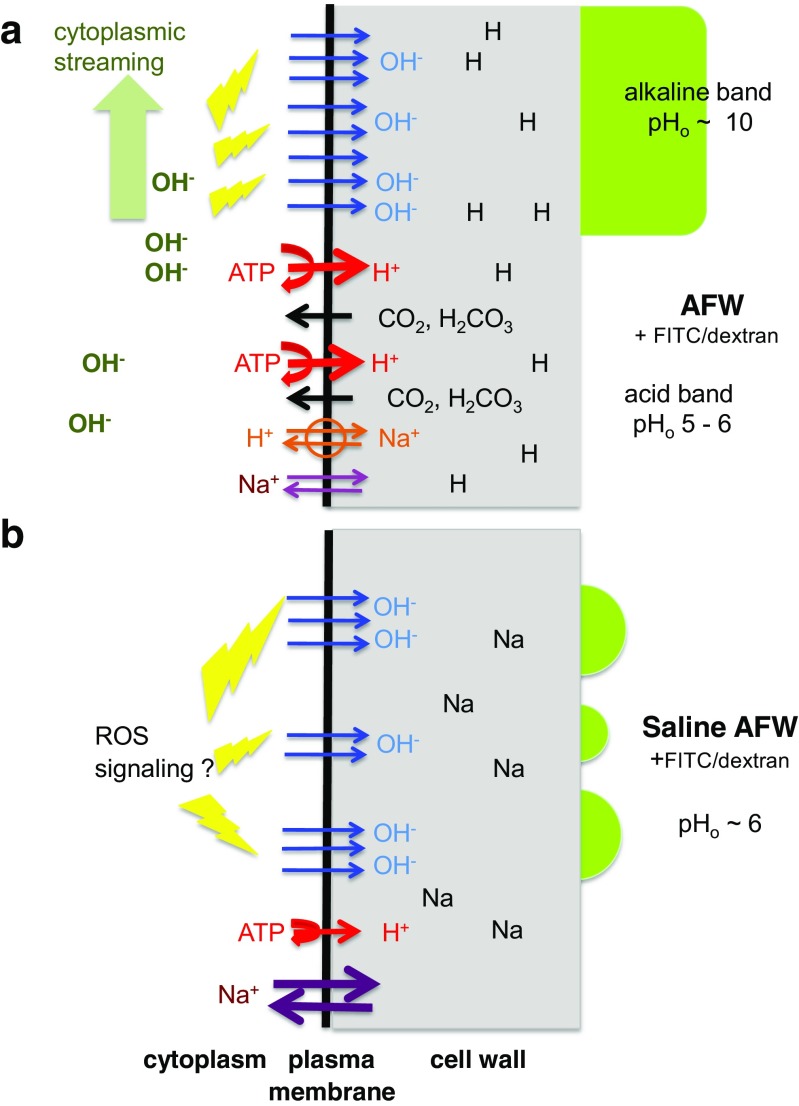
The schematic diagram for activation of OH^−^ channels (**a**) in AFW during pH banding (**b**) in saline AFW. **a** In AFW and in the light, proton pumps (red arrows) acidify the vicinity of the membrane. DIC (dissolved inorganic carbon) balance tips toward CO_2_ and H_2_CO_3_. The neutral molecules, CO_2_ and possibly H_2_CO_3_, can cross the lipid bilayer (black arrows). The byproduct of carbon entering the Calvin cycle in the chloroplasts is OH^−^ (see Fig. 11.6 in Beilby and Bisson 2012). OH^−^ accumulates in the streaming cytoplasm (light green arrow) under the acid band and eventually activates (yellow lightning bolts) the OH^−^ channels (blue arrows). This zone becomes the alkaline band, where OH^−^ is excreted until pH drops before the next acid band upstream (where it starts to rise again). The alkaline band is visualized by FITC, fluorescing at high pH (bright green color). The attached dextran stops the FITC entering the cell wall space. Na^+^ and other cations are imported into the cytoplasm by non-selective cation channels (purple arrows). Excess Na^+^ is exported by Na^+^/H^+^ antiporter (orange arrows). There are other transporters activated by the inward H^+^ gradient, but these were omitted for simplicity. As the cation concentration in the medium is low, some of the negative fixed charges on the wall matrix are neutralized by H^+^ (“H” in the wall space). For detailed review of the banding system, as well as the various transporters, see Chap. 2 of Beilby and Casanova (2014). **b** In saline AFW, greater concentration of Na^+^ in the medium increases the inflow through non-selective cation channels (thicker purple arrows, Shepherd et al. 2008; Beilby and Shepherd 2001). After longer exposures to saline, the proton pump becomes gradually inhibited (thinner red arrow, Shepherd et al. 2008), the membrane PD approaches *E*_OH_ (Beilby and Al Khazaaly 2009) and the H^+^ powered transporters no longer work. The OH^−^ channels might be activated by ROS signaling (yellow lightning bolts), independent of cytoplasmic pH or streaming (Al Khazaaly et al. 2009; Eremin et al. 2013). Initially, the activation is transient, as the negative membrane PD is less favorable to channel open states (see G/V characteristics 3 in Fig. 1f). With exposure to saline, the membrane PD becomes more positive with diminishing pump action (Shepherd et al. 2008) and the OH^−^ channels open for longer intervals (Al Khazaaly et al. 2009; Beilby and Al Khazaaly 2009). With the greater concentration of Na^+^ in the medium, the H^+^ ions neutralizing the fixed wall charges are replaced by Na^+^ in about 10 min of saline exposure (“Na” in the wall space; Holland and Barr 1982). See text for discussion on permeation of OH^−^ through the cell wall space

It is possible that Na^+^ in saline AFW exchange for H^+^, bound to the negative fixed charges in the cell wall (Fig. 6b). The plant cell walls have a high cation-binding capacity (Metraux et al. 1980; Holland and Barr 1982; Meychik et al. 2003; Meychik et al. 2014). Holland and Barr (1982) found that 20 mM KCl induced rapid release of protons from the cell wall. The rate of release increased with the pH, and at pH 7.9, all the available bound protons were extruded from the cell wall within 10 min. Therefore, the H^+^ ions, replaced by excess Na^+^ in the wall, most probably neutralized the initial “spits” of OH^−^. This hypothesis will be explored in future experiments with FITC conjugated with smaller dextran molecules that allow access to cell wall space. In the external medium, the OH^−^ is likely to disperse and/or recombine with H^+^.

Beilby and Al Khazaaly (2009) hypothesized that the transient opening of the H^+^/OH^−^ channels (seen as the typical spiky electrical noise in Fig. 1a–c) is due to the membrane PD being too negative to favor long open states (see I/V and G/V characteristics for the channels in Fig. 1e and f). The fluctuating spots of bright FITC-dextran fluorescence (Figs. 3 and 4, Suppl. Videos 1a and b) are likely to correspond to the larger PD spikes (the smaller “OH^−^ spits” might disperse inside the cell wall). This hypothesis can be checked quantitatively. Let us assume a typical alkaline spot with a diameter of 30 μm on the cell wall surface, hemispherical shape (volume *V* = 7.65 ∗ 10^−15^ L), and local pH 9 (OH^−^ concentration *c* = 10^−5^ mol/L as pOH = − log[OH^−^] = 14 − pH = 5). The total number *N* of hydroxide ions in the bright spot becomes *N* = *c* ∗ *N*_A_ = 4.6 ∗ 10^4^ (Avogadro’s number *N*_A_ = 6.02 ∗ 10^23^ ions/mol). These *N* ions crossed the plasma membrane within a time of 10 seconds (approx. time of bright spot occurrence), passing through the open OH^−^ channels localized on a finite small membrane area. An ionic current is thus generated *i* = 4.6 ∗ 10^4^ ∗ 1.6 ∗ 10^−19^ ∗ 10^−1^ = 7.36 ∗ 10^−16^ A (1.6 ∗ 10^−19^ C is the elementary electronic charge). Generally, the number of ion channels in a cell plasma membrane is known to be of the order of a few hundreds/μm^2^. In an eosinophil cell, for example, there are 125–200 voltage-gated H^+^ channels/μm^2^ (DeCoursey 2003) and the typical single channel conductance is extremely low: 15 ∗ 10^−15^ S at physiological pH and 20 °C (DeCoursey 2013). If we consider a large spike of 10 mV in the PD noise (Fig. 1b and c), the total channel conductance of *g* = *i*/PD = 7.36 ∗ 10^−16^ A / 10^−2^ V = 73.6 ∗ 10^−15^ S corresponds to ~ 5 open channels. The open probability of the OH^−^ channels at the time of the transient spot appearance is quite low, so these five channels on a membrane patch of the order of 1 μm^2^ corresponds to 2–5% of the channels being open. The spit of OH^−^ will acidify the cytoplasm beneath the patch, which in turn might activate nearby H^+^ pumps and more H^+^ in the wall contributes to rapid spot disappearance.

After longer exposures to saline AFW, the membrane PD drops to approximately − 100 mV (I/V 2 in Fig. 1d) and the conditions are better for the H^+^/OH^−^ channels to remain open and to contribute to the I/V characteristics (I/V 3, Fig. 1d; Beilby and Al Khazaaly 2009). The high pH spots become more fixed in space (Fig. 4). A positive feedback may be involved here: the longer the channels are activated, the more OH^−^ accumulates in the wall raising the local pH, which in turn activates more channels. To model the I/V characteristics of such depolarized cells, the OH^−^ conductance of the cell reached up to 3 S m^−2^ (Fig. 1f). Dividing by the unitary channel conductance (DeCoursey 2013), we obtain ~ 200 channels/μm^2^, similar to animal systems and a reasonable number of channels/μm^2^ in plants.

In the electrophysiological experiments, the cells remained hyperpolarized in sorbitol AFW, the membrane PD was often less noisy than even in AFW, and the I/V characteristics were pump-dominated (curve 1 in Fig. 1d; Shepherd et al. 2008; Al Khazaaly et al. 2009; Beilby and Al Khazaaly 2009). These findings were confirmed by the pH imaging: no transient bright pH spots were observed and the pH banding was not affected in accordance to earlier findings that turgor reduction of up to 210 mOsmol did not disturb the position of the OH^−^ bands in *Chara* (Lucas and Alexander 1981). Therefore, the transient H^+^/OH^−^ channel activation is caused by the high salt component of the salinity stress.

Some control cells in AFW did exhibit high pH spots. These spots appeared prior to the formation of alkaline band/s once the cell recovered from being manipulated into the experimental chamber. Supplementary Fig. 1 shows such spot amalgamation into a large alkaline band. Thus, our experiments captured an interesting aspect of the *Chara* H^+^/OH^−^ channels: an element of self-organization, where the small numbers of activated channels grow under the right conditions. Bulychev et al. (2003) observed similar spot amalgamation at the time of the band formation. Permanent high pH spots were occasionally seen at the sites where additional cell wall material had been or is still being deposited beneath epiphytes (Suppl. Fig. 2; Foissner and Wasteneys 2012). However, the dynamics of both the manipulation-induced pH spots, initiating the alkaline bands, and the wound wall spots were different from those observed in the salt stressed cells.

The disappearance of the alkaline band in the saline AFW (Suppl. Fig. 1) seems counter-intuitive, as salinity clearly stimulates the H^+^/OH^−^ channel activation. It appears that different mechanisms are involved on different time scales. The progressive collapse of the banding pattern is caused by the gradual inactivation of the proton pump (see transition from I/V curves 1 to 2 in Fig. 1d and e and the diagram of the banding scheme in Fig. 6a), which makes the acid band increasingly less acid. This local increase in pH results in less CO_2_ (and H_2_CO_3_) being transported into the chloroplasts for carbon fixation and less OH^−^ being generated in the cytoplasm. It is the rise of the cytoplasmic pH under the normal conditions, which is thought to activate the H^+^/OH^−^ channels in the alkaline bands (see Fig. 11.6 of Beilby and Bisson 2012 and Fig. 6a). Consequently, there can be no alkaline bands without the carbon concentrating action of the acid bands. The activation of H^+^/OH^−^ channels by saline AFW seems to be by another mechanism. Ca^2+^ signaling is not involved, as the cytoplasmic streaming was not affected by the appearance of alkaline spots (for review of Ca^2+^ concentration effects on the cytoplasmic streaming see Chap. 4 of Beilby and Casanova 2014). As described in the “Introduction”, ROS signaling is implicated (Eremin et al. 2013; Dodonova et al. 2010; Li et al. 2007; Beilby et al. 2014). The random distribution of the alkaline spots over the cell surface (Figs. 3, 4, and 6b, Suppl. Videos 1a and b) might be prompted by oxidative events in different parts of the giant cell. Such possibility will be addressed in future experiments.

In the electrophysiological experiments, the voltage clamping drives large currents across the cell membrane, adding to further stress for the cell. The typical survival in saline AFW was 2–3 h (Shepherd et al. 2008; Beilby and Al Khazaaly 2009). All the cells exhibited initial depolarization upon exposure to saline AFW (see Fig. 1a). Some cells then recovered and remained at PDs more negative than − 100 mV for many minutes with the proton pump activity diminishing slowly (Fig. 1a), while others continued to depolarize to − 100 mV and above (Al Khazaaly et al. 2009; Beilby and Al Khazaaly 2009). The reasons for this variability are thought to be the cell age (younger cells being more vulnerable) and seasonal effects. However, in the present experiments, the alkaline band/s vanished in minutes, once the cells were exposed to saline AFW (Suppl. Fig. 1). As with the early alkaline spots, the H^+^ release from the alkaline cell wall regions would quickly neutralize the OH^−^ in the alkaline bands. The drop of the high external pH in the alkaline band was likely to contribute to OH^−^ channel closure, but a weaker band would re-appear in cells where the proton pump was slow to inactivate.

The literature on the H^+^/OH^−^ channels in the animal kingdom was helpful, suggesting the Zn^2+^ blocking and showing similarities in channel activation by increase in ΔpH (pH_o_–pH_i_), the membrane PD depolarization, and increase in ROS levels (DeCoursey 2003, 2013; Musset et al. 2010; DeCoursey and Hosler 2014; Wu 2014). However, the sequence data of *Chara* and, indeed, of higher plants lack the homologies to animal H^+^ channels. There are several other putative plant VGHC sequences available in public databases, but again no homologous forms were detected in *Chara*. We screened two different cDNA databases of *C. australis*, obtained by the next generation sequencing, and performed nested, as well as regular PCRs using degenerated primers, which were designed by aligning homologous regions of different plant VGHCs, without success (Suppl. Fig. 4). Furthermore, Stefan Rensing screened the *C. braunii* genome without detecting any VGHCs (in preparation). However, he found a protein similar to voltage-gated Ca^2+^ channels, which confirms the existence of voltage-gated proteins in *Chara*. These data raise the question if *Chara* uses different H^+^/OH^−^ transport proteins, compared to those sequenced in animals and land plants. Such possibility is supported by many protein structures that involve narrow water pores or can sustain proton/hole hopping without water (DeCoursey and Hosler 2014).

There are some similarities between *Chara* and land plant genomes: we detected a member of the cation/H^+^ exchanger superfamily in *Chara*, which shares 40% amino acid identities with *A. thaliana* CHX17 (At4g23700) (Suppl. Fig. 5). AtCHX17 is involved in regulation of pH homeostasis and is associated with the plasma membrane and post-Golgi compartments (Cellier et al. 2004; Chanroja et al. 2013). Furthermore, we detected several partial proteins sequences that showed 30–40% amino acid identity to different voltage-gated K^+^ channels of *A. thaliana*.

Could H^+^ or OH^−^ permeate through non-selective cation or anion channels? The joint results from electrophysiology and fluorescence microscopy make this possibility rather unlikely. The non-selective channel would have to become very selective in the alkaline band, upon exposure to high pH and at the time of saline stress. In the last case, the selectivity would have to change transiently, and later with greater duration, on small patches of membrane.

How is the role of H^+^/OH^−^ channels in *C. australis* salt sensitivity relevant to land plants in general? Even if the land plants, sea algae, and animal H^+^/OH^−^ channel proteins are different, these channels fulfill similar role of controlling pH in the cell compartments and multicellular organs. If salinity inactivates the pump and opens the H^+^/OH^−^ channels in the subapical zone of roots, the normal root function in higher plants will be disrupted (Raven 1991). Inward currents at membrane PDs more positive than *E*_K_ and *E*_Cl_ and salinity-induced noise were observed in salt-stressed wheat root protoplasts (Tyerman et al. 1997) and the H^+^/OH^−^ I/V characteristics could be modeled (Tyerman et al. 2001). Consequently, these channels may play an important role in salinity stress in salt-sensitive land plants but have been mostly ignored up to date.

## Electronic supplementary material

Supplementary Fig. 1 The time course of pH band formation visualized by FITC-dextran 70 dissolved in AFW. **a** Note that in this cell, smaller spots give rise to a large alkaline band. **b** Disappearance of the pH band after placing the cell into saline AFW. Bar is 250 μm for all images. (PPTX 1026 kb)

Supplementary Fig. 2 The alkaline spot at a wound wall in AFW. **a** FITC-dextran 70 image, **b** corresponding bright field image, **c** merged image, **d** wound wall at higher magnification. The arrow in **b** indicates the position of the wound wall which had displaced the cortical chloroplasts, the arrow in **d** indicates the wound wall. Bars are 250 μm (**a**, **b**, **c**) and 20 μm (**d**). (PPTX 654 kb)

Supplementary Fig. 3 The inhibitory effect of ZnCl_2_ on pH banding. **a** Visualization of an alkaline band by FITC-dextran 70 dissolved in AFW, **b** the band suppression by addition of 1 mM ZnCl_2_, and **c** the corresponding bright field image. Bars are 250 μm. (PPTX 294 kb)

Supplementary Fig. 4 **a** Multiple protein sequence alignment of different predicted voltage-gated hydrogen channel sequences (*Nicotiana sylvestris* XP_009803685, *N. tabaccum* XP_00961780, *Fragaria vesca* XP_004298021, *Glycine soja* KHN29519, *Medicago truncatula* XP_003621655, *Klebsormidium flaccidum* GAQ80331, *Selaginella moellendorfii* XP_00298883, *Marchantia polymorpha* OAE32766, *Physcomitrella patens* (XP_001767834). The red box marks the most homologous, overlapping part, which was used as a template to design degenerated primers. **b** Sequence alignment of the homologous part (red box in **a**) of all available corresponding mRNAs (obtained from NCBI) of the protein (JPEG 239 kb)

High-resolution image (TIFF 32993 kb)

Supplementary Fig. 5 Sequence alignments of cation/H^+^ exchanger AtCHX17 and homologous proteins. Multiple protein sequence alignment of cation/H^+^ exchanger proteins including *Arabidopsis thaliana* AtCHX17 (At4g23700) and homologous proteins from *C. australis* (CaCHX accession number: KY751909), *Selaginella moellendorfii* (XP_002974580) and *Marchantia polymorpha* (OAE27085). The green box marks the cation/H^+^ exchanger domain (IPR006153) according to Interpro (Mitchell et al. 2015). (JPEG 335 kb)

High-resolution image (TIFF 36109 kb)

Supplementary Videos 1 **a** and **b** The time series of alkaline spots appearing at the surface of cells treated with 50 mM NaCl containing FITC-dextran 70. **a** The video shows the FITC fluorescence and starts after 77 min treatment. **b** Video of another cell starting after 31 min treatment; the fluorescence images are merged with the bright field images. Bars are 200 μm. (AVI 1324 kb)

(AVI 2052 kb)

Supplementary Table 1 Primer list. List of degenerated primers used for the attempt of cloning of voltage gated hydrogen channels (VGHC) in *Chara australis*. (DOCX 13 kb)
