# Supplementary figures and images for: Surface pH changes suggest a role for H+/OH− channels in salinity response of Chara australis
Source: Protoplasma. 2017 Dec 15;255(3):851–62. doi: 10.1007/s00709-017-1191-z (PMC5904247; doi:10.1007/s00709-017-1191-z)

## Slide 1
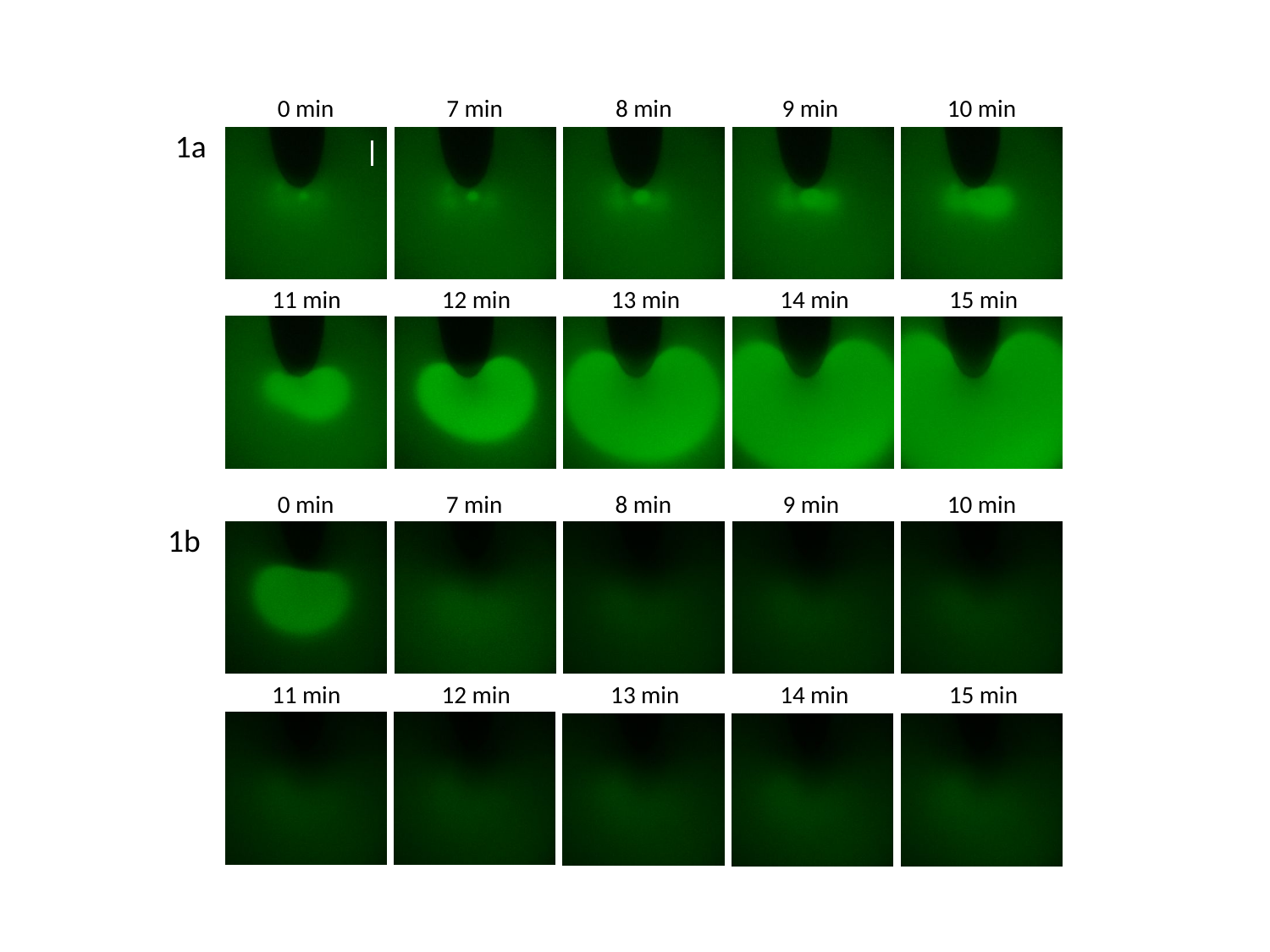

0 min
7 min
8 min
9 min
10 min
1a
11 min
12 min
13 min
14 min
15 min
0 min
7 min
8 min
9 min
10 min
1b
11 min
12 min
13 min
14 min
15 min

Supplement: Supplementary file 1 — The time course of pH band formation visualized by FITC-dextran 70 dissolved in AFW. a Note that in this cell, smaller spots give rise to a large alkaline band. b Disappearance of the pH band after placing the cell into saline AFW. Bar is 250 μm for all images. (PPTX 1026 kb) [file 709_2017_1191_MOESM1_ESM.pptx]

## Slide 1
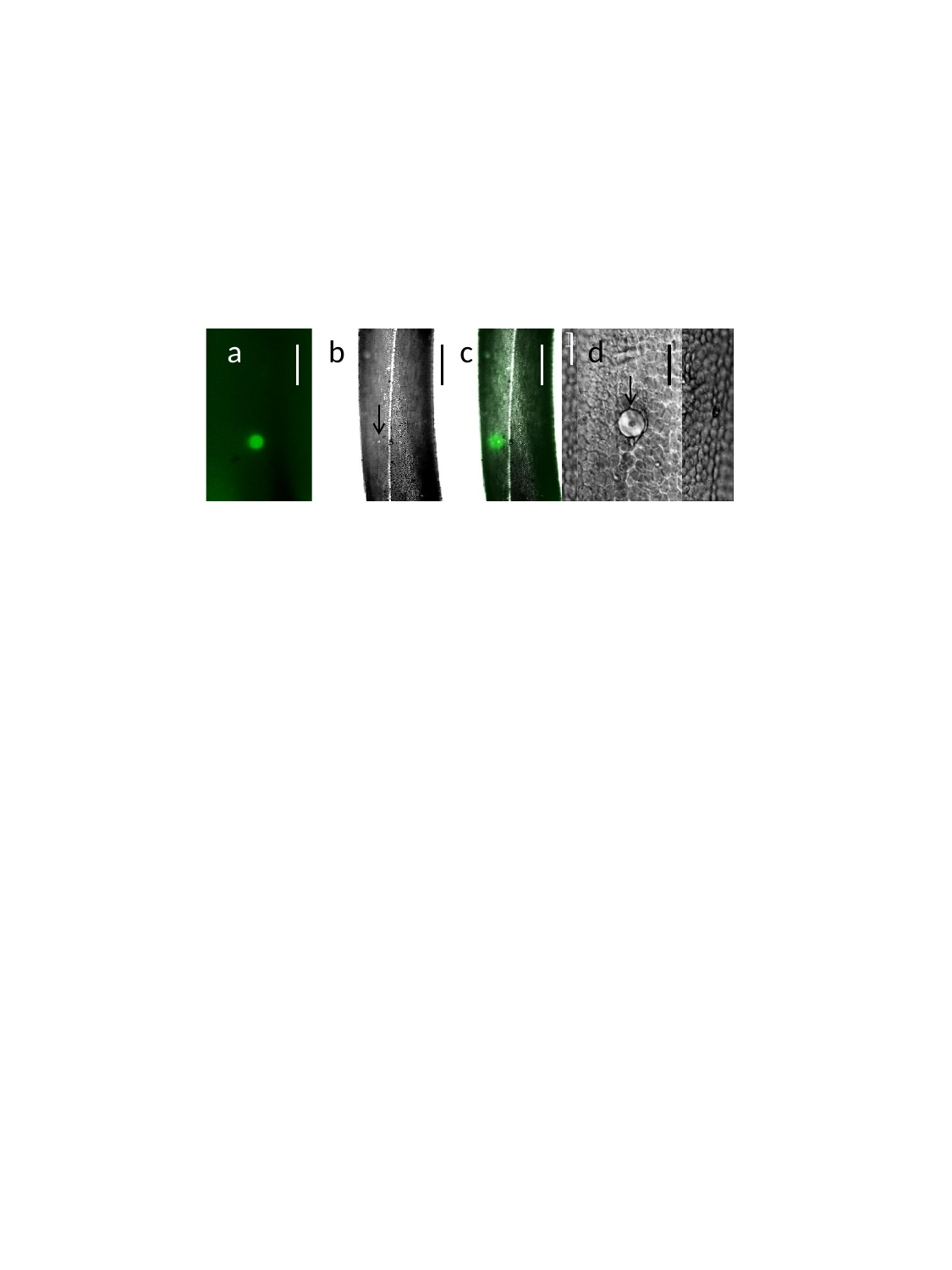

a b c d

Supplement: Supplementary file 2 — The alkaline spot at a wound wall in AFW. a FITC-dextran 70 image, b corresponding bright field image, c merged image, d wound wall at higher magnification. The arrow in b indicates the position of the wound wall which had displaced the cortical chloroplasts, the arrow in d indicates the wound wall. Bars are 250 μm (a, b, c) and 20 μm (d). (PPTX 654 kb) [file 709_2017_1191_MOESM2_ESM.pptx]

## Slide 1
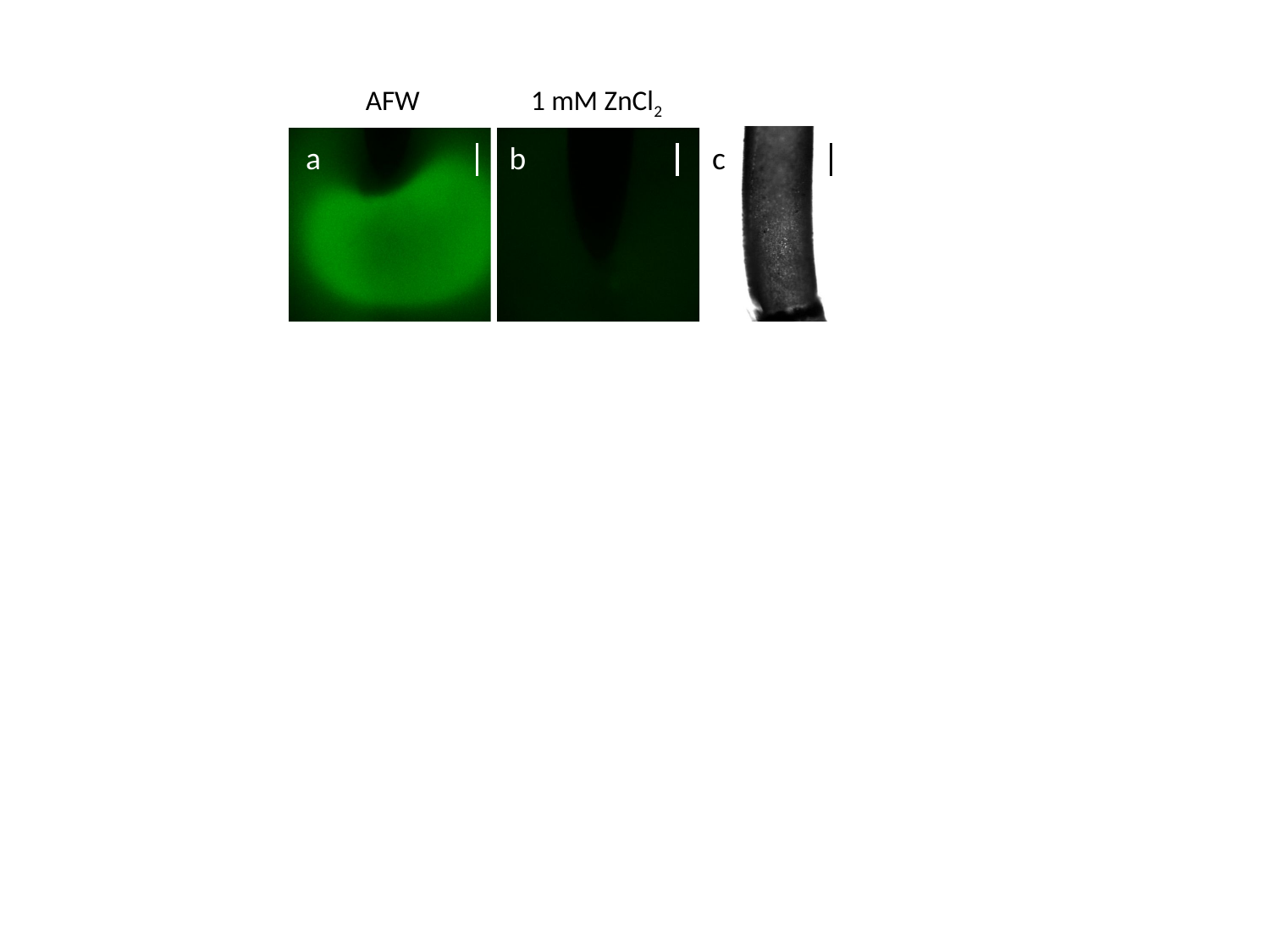

AFW
1 mM ZnCl2
a
b
c

Supplement: Supplementary file 3 — The inhibitory effect of ZnCl2 on pH banding. a Visualization of an alkaline band by FITC-dextran 70 dissolved in AFW, b the band suppression by addition of 1 mM ZnCl2, and c the corresponding bright field image. Bars are 250 μm. (PPTX 294 kb) [file 709_2017_1191_MOESM3_ESM.pptx]

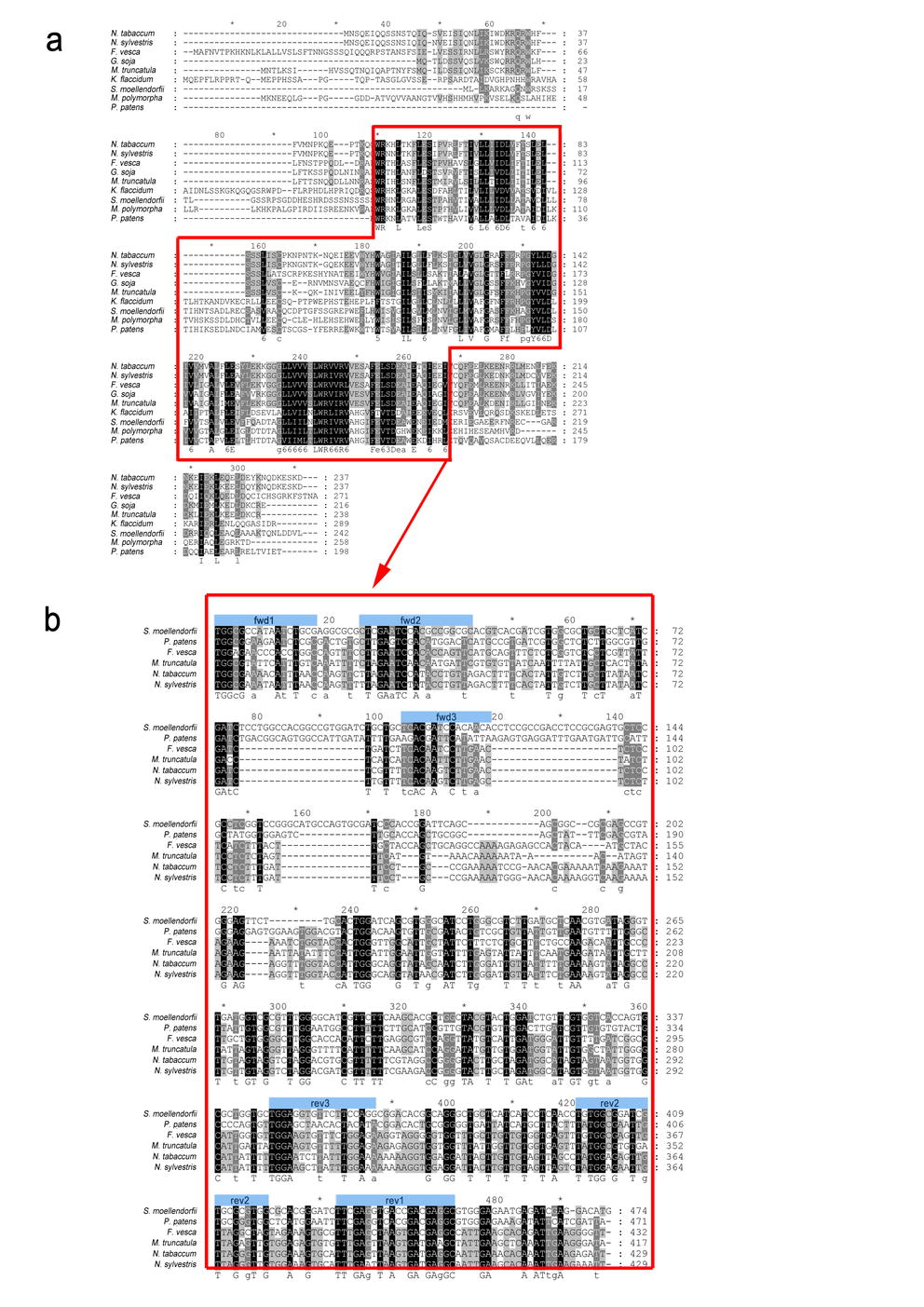

Supplement: Supplementary file 4 — a Multiple protein sequence alignment of different predicted voltage-gated hydrogen channel sequences (Nicotiana sylvestris XP_009803685, N. tabaccum XP_00961780, Fragaria vesca XP_004298021, Glycine soja KHN29519, Medicago truncatula XP_003621655, Klebsormidium flaccidum GAQ80331, Selaginella moellendorfii XP_00298883, Marchantia polymorpha OAE32766, Physcomitrella patens (XP_001767834). The red box marks the most homologous, overlapping part, which was used as a template to design degenerated primers. b Sequence alignment of the homologous part (red box in a) of all available corresponding mRNAs (obtained from NCBI) of the protein (JPEG 239 kb) [file 709_2017_1191_Fig7_ESM.jpg]

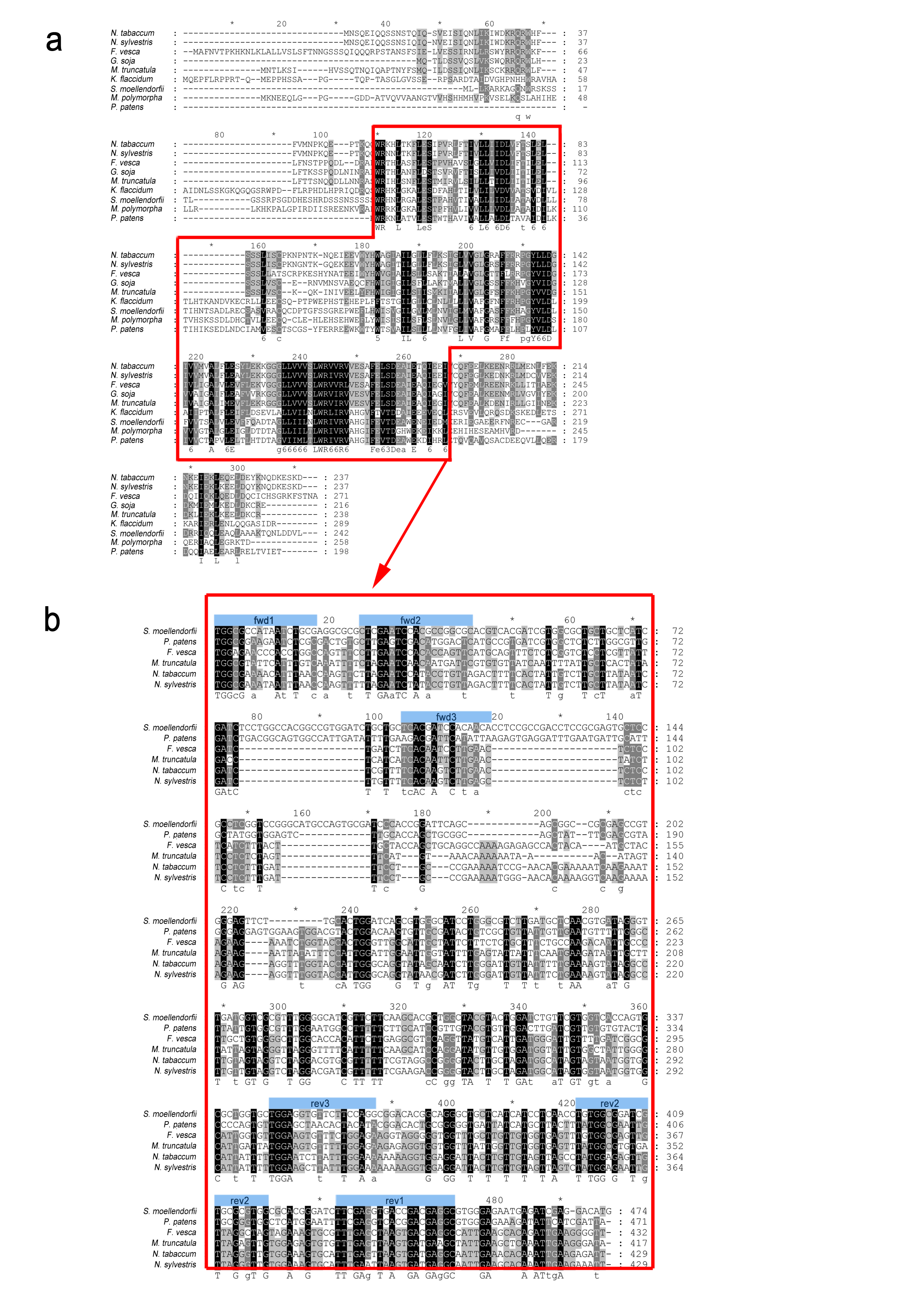

Supplement: Supplementary file 5 — High-resolution image (TIFF 32993 kb) [file 709_2017_1191_MOESM4_ESM.tif]

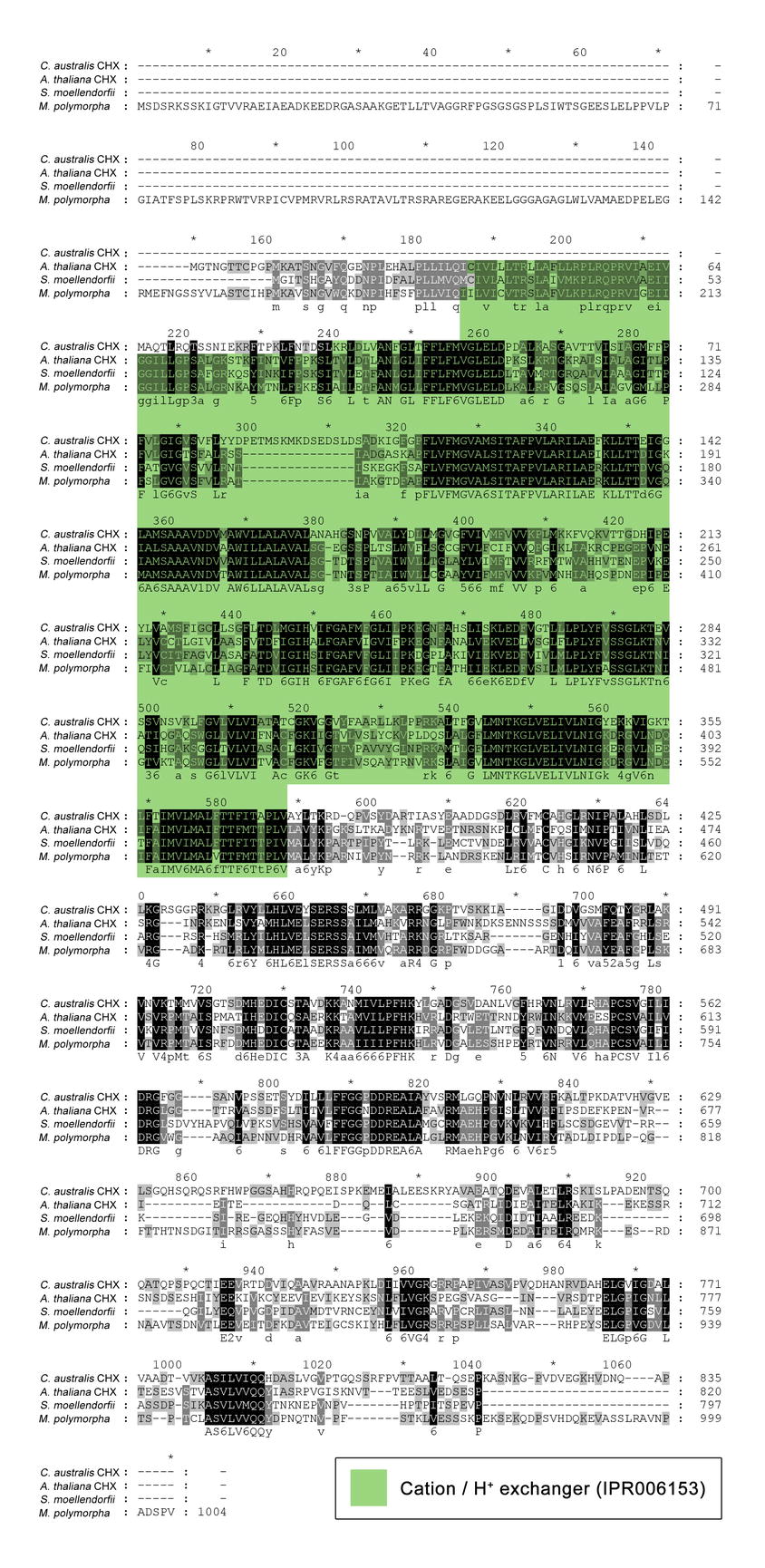

Supplement: Supplementary file 6 — Sequence alignments of cation/H+ exchanger AtCHX17 and homologous proteins. Multiple protein sequence alignment of cation/H+ exchanger proteins including Arabidopsis thaliana AtCHX17 (At4g23700) and homologous proteins from C. australis (CaCHX accession number: KY751909), Selaginella moellendorfii (XP_002974580) and Marchantia polymorpha (OAE27085). The green box marks the cation/H+ exchanger domain (IPR006153) according to Interpro (Mitchell et al. 2015). (JPEG 335 kb) [file 709_2017_1191_Fig8_ESM.jpg]

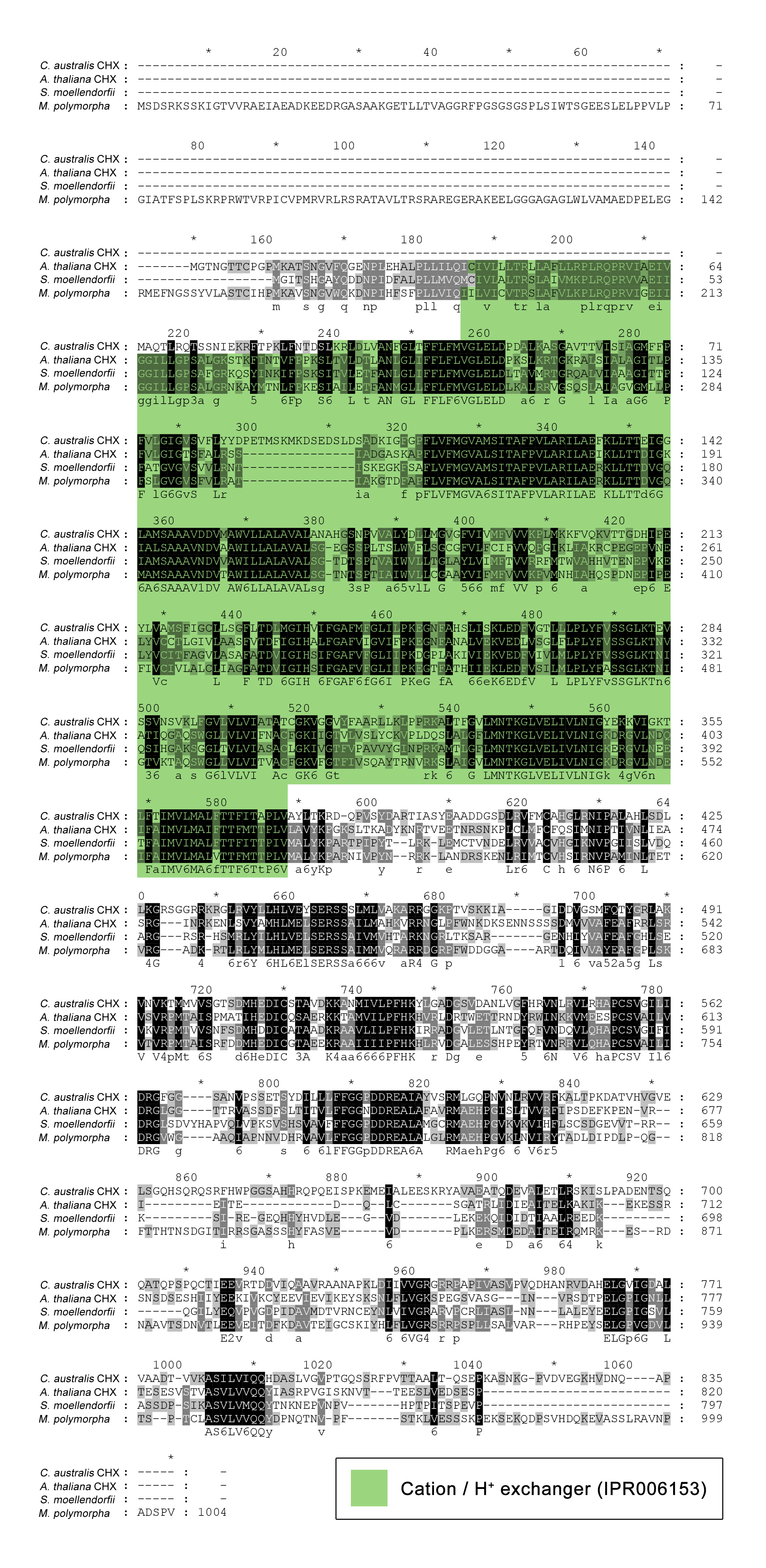

Supplement: Supplementary file 7 — High-resolution image (TIFF 36109 kb) [file 709_2017_1191_MOESM5_ESM.tif]
