# Supplementary material for: Surface pH changes suggest a role for H+/OH− channels in salinity response of Chara australis
Source: Protoplasma. 2017 Dec 15;255(3):851–62. doi: 10.1007/s00709-017-1191-z (PMC5904247; doi:10.1007/s00709-017-1191-z)
Supplement: Supplementary file 10 — Primer list. List of degenerated primers used for the attempt of cloning of voltage gated hydrogen channels (VGHC) in Chara australis. (DOCX 13 kb) [file 709_2017_1191_MOESM8_ESM.docx]

| **Suppl. Table 1** Primer list. List of degenerated primers used for the attempt of cloning of voltage gated hydrogen channels **(VGHC)** in *Chara australis*. | |
| --- | --- |
| **name** | **sequence** |
| VGHC_deg_fwd1 | 5'-TGGMGNMHNMAYYTVNS-3' |
| VGHC_deg_fwd2 | 5'-YTHGARTCNAYRHBDRYKM-3' |
| VGHC_deg_fwd3 | 5'-WSACRAKYCWYRWNM-3' |
|  |  |
| VGHC_deg_rev1 | 5'-GCYTCRTCVSTBAVYTCRAA-3' |
| VGHC_deg_rev2 | 5'-CHASBCKMAYVAYHCKCCAYA-3' |
| VGHC_deg_rev3 | 5'-YHTSBARDDWHRVYTCCA-3' |
